# Clinical outcomes of combined catheter ablation and left atrial appendage closure in elderly patients with nonvalvular atrial fibrillation

**DOI:** 10.1002/clc.24169

**Published:** 2023-10-07

**Authors:** Xiaohong Fei, Binhao Wang, Huimin Chu, Guohua Fu, Yibo Yu, Mingjun Feng, Xianfeng Du, Jing Liu

**Affiliations:** ^1^ Arrhythmia Center, the First Affiliated Hospital of Ningbo University Ningbo China; ^2^ Key Laboratory of Precision Medicine for Atherosclerotic Diseases of Zhejiang Province Ningbo Zhejiang Province China

**Keywords:** atrial fibrillation, catheter ablation, combined procedure, elderly, left atrial appendage closure

## Abstract

**Background:**

Catheter ablation (CA) combined with left atrial appendage closure (LAAC) has emerged as a promising method to relieve symptoms while reducing the incidence of stroke in selected high‐risk patients with atrial fibrillation (AF).

**Hypothesis:**

We aimed to investigate the clinical outcomes of combined CA and LAAC in elderly patients.

**Methods:**

A total of 316 patients with symptomatic drug‐refractory AF who underwent combined CA and LAAC between January 2016 and December 2020 were retrospectively included. Baseline characteristics, periprocedural complications, and clinical events during follow‐up were recorded and compared between patients aged ≥ 75 years (*n* = 66) and <75 years (*n* = 250).

**Results:**

Pulmonary vein isolation and satisfactory LAAC were achieved in all patients. No patients experienced death or stroke/transient ischemic stroke periprocedurally. After a median follow‐up of 12.2 (6.7−24.4) months and 11.9 (5.5−23.6) months, the rate of sinus rhythm maintenance was comparable between the two groups (≥75 years: 78.8% vs. <75 years: 80.8%; log‐rank test, *p* = 0.674). The median follow‐up periods for clinical outcomes were 27.9 (9.3−44.8) months and 25.2 (10.8−45.7) months, respectively. In patients aged ≥ 75 years, one suffered ischemic stroke, and one experienced major bleeding event. In patients aged < 75 years, four had ischemic stroke, and eight had major bleeding events. Two patients aged < 75 years died during follow‐up, while none of the patients aged ≥ 75 years died.

**Conclusions:**

Combining CA and LAAC was feasible, safe and effective in elderly patients with AF.

## Graphical abstract

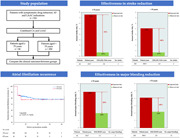

## 1 INTRODUCTION

Atrial fibrillation (AF) is the most common type of arrhythmia, with an increasing incidence and prevalence in patients with advancing age.^1^ Treating elderly patients with AF remains a major therapeutic challenge for physicians because antiarrhythmic drugs (AADs) are not effective, and they pose significant risks. Catheter ablation (CA) has been proven to be an effective treatment for symptomatic AF and decreases the risk of AF recurrence compared to AAD therapy in elderly patients.^2^, ^3^, ^4^

Patients with AF have an increased risk of ischemic stroke, which is approximately 5‐fold higher than that of the general population.^5^ Current European guidelines recommend oral anticoagulation (OAC) for patients with AF and a high risk of stroke and without contraindications to OACs.^1^ Although OAC therapy is effective in preventing ischemic stroke in elderly individuals, it also imposes a significant risk of major bleeding complications.^6^ Currently, left atrial appendage closure (LAAC) is considered for stroke prevention in patients with AF and contraindications to long‐term use of OACs.

Combining CA and LAAC in a single procedure has recently gained increasing attention and could simultaneously relieve AF symptoms while reducing the incidence of stroke in selected high‐risk patients. Previous studies have demonstrated a high procedural success rate with a relatively low complication rate of the combined procedure as well as satisfactory follow‐up results were obtained.^7^ However, data on combined CA and LAAC in the elderly patient population are limited. The aim of this study was to report the clinical outcomes of combined CA and LAAC in elderly patients with AF.

## 2 METHODS

### 2.1 Study population

Patients with symptomatic drug‐refractory AF from the Arrhythmia Center of Ningbo First Hospital between January 2016 and December 2020 were retrospectively included. Thromboembolism and bleeding risk were defined according to the CHA_2_DS_2_‐VASc and HAS‐BLED scores, respectively.^8^, ^9^ Patients were included according to the following criteria: (1) age > 18 years; (2) nonvalvular AF refractory to AADs; (3) CHA_2_DS_2_‐VASc score ≥ 2 in men and ≥3 in women and/or HBS‐BLED score ≥ 3, or contraindications for long‐term OAC therapy (e.g., patients with bleeding events or thromboembolic events under OAC or intolerance); or unwillingness to receive long‐term OAC therapy. The study protocol was approved by the Ethics Committees of the First Affiliated Hospital of Ningbo University and complied with the Declaration of Helsinki. All participants were fully informed, and written informed consent was obtained.

### 2.2 Preprocedural assessment

Transesophageal echocardiography (TEE) was performed 24 h before the procedure to exclude thrombi within the left atrium (LA) and left atrial appendage (LAA). The LAA orifice diameter was measured, and transthoracic echocardiography was conducted to measure the LA diameter and left ventricular ejection fraction (LVEF). AAD treatment was stopped five half‐lives before the procedure. Warfarin at a therapeutic international normalized ratio (INR) was continued uninterrupted, while nonvitamin K antagonist oral anticoagulants were ceased 12−24 h preprocedurally.

### 2.3 Combined CA and LAAC procedure

Patients were placed under deep sedation during the procedure. For the CA procedure, a circular mapping catheter and an irrigated tip ablation catheter were utilized for mapping and ablation, respectively. The ablation settings used were 40 W/43°C/25 mL per min for the roof and anterior walls and 35 W/43°C/17 mL per minute for the posterior and inferior walls, with a target force of 5–15 g. The ablation was guided by ablation index (AI) or lesion size index (LSI). The target AI values were 500‐550 for the roof and anterior walls and 350–400 for the posterior and inferior walls, respectively. The target LSI values were 4.5–5.5 for the roof and anterior walls and 4.0–4.5 for the posterior and inferior walls, respectively. Circumferential pulmonary vein isolation (PVI) was performed in patients with AF. Additional linear lesions and/or complex fractionated atrial electrograms were targeted if necessary. For the LAAC procedure, TEE or intracardiac echocardiography (ICE) was applied to guide device implantation. The type (including Watchman, the Amplatzer Cardiac Plug, and LAmbre) and size of the occluder was selected by the operator according to the TEE/ICE measurements and LAA angiogram results. The device was advanced into the LAA through the delivery sheath and deployed via sheath retraction. The device was released after confirmation of an adequate position [no or minimal peridevice leakage (PDL) ≤ 5 mm by TEE/ICE] and a tug test.

Major periprocedural adverse events included death, stroke, transient ischemic attack (TIA), and major bleeding events. Minor periprocedural complications included minor bleeding or access site complications (e.g., arteriovenous fistula, femoral hematoma, and pseudoaneurysm) without the need for further intervention. Major bleeding was defined according to the Bleeding Academic Research Consortium criteria (type 3 or higher).^10^

### 2.4 Follow‐up

AAD and OAC therapy (warfarin with an INR of 2−3, rivaroxaban 15−20 mg once daily, or dabigatran 110−150 mg twice daily) was recommended for 3 months following the procedure. Dual antiplatelet therapy (aspirin 100 mg and clopidogrel 75 mg once daily) was prescribed between 3 and 6 months, and single antiplatelet therapy (aspirin 100 mg or clopidogrel 75 mg once daily) was prescribed after 6 months. TEE was performed to assess device occlusion, PDL and device‐related thrombosis (DRT) at 45 days and 6 months. OACs were reinitiated, and TEE reassessment was recommended if DRT or PDL > 5 mm was detected during follow‐up until the thrombus was completely resolved.

Clinical follow‐up for AF recurrence was performed at 3, 6, and 12 months using 24 h Holter monitoring. AF recurrence was defined as documented AF that lasted at least 30 s after a 3‐month blanking period. Clinical outcomes included death, ischemic stroke, TIA, and bleeding events (major or minor). All patients were monitored by follow‐up TEE, clinic visits, and phone calls by our research staff. Stroke was defined as the onset of a new neurologic deficit persisting for >24 h. It was confirmed by cerebral magnetic resonance imaging or CT by at least two radiologists or neurologists. If the duration of the deficit was <24 h, it was defined as a TIA.

### 2.5 Statistical analysis

The study population was divided into two groups based on age: ≥ 75 years and <75 years. Normally distributed continuous variables are expressed as the mean ± standard deviation, while the median (interquartile range) is used for variables with a skewed distribution. Categorical variables are expressed as absolute numbers (percentages). Continuous variables were compared using the *t* test and Mann‒Whitney *U* test for normally and nonnormally distributed data, respectively. Categorical variables were compared using the *χ* ^2^ test or Fisher's exact test where appropriate.

Device efficacy for preventing stroke and reducing major bleeding events was assessed by comparing the actual event rate (including both periprocedural and follow‐up events) with the predicted event rate according to the CHA_2_DS_2_‐VASc score and HAS‐BLED score. The individual patient annual risk was recorded, and the average annual risk was calculated. The total number of stroke or major bleeding events was divided by the total patient‐years of follow‐up and multiplied by 100 to obtain the actual annual rate. Stroke and bleeding reduction were calculated as follows: (estimated—actual event rate)/estimated event rate.

Survivor functions were estimated using the Kaplan–Meier method to assess the cumulative event‐free curves of AF for each group and statistically evaluated using a log‐rank test of trend. Statistical analyses were performed with SPSS 19.0 (IBM), and a *p* < 0.05 was considered statistically significant.

## 3 RESULTS

### 3.1 Patient characteristics

Patient characteristics are shown in Table 1. There were 66 and 250 patients aged ≥ 75 years and <75 years, respectively. The percentage of congestive heart failure was higher in patients aged ≥ 75 years (22.7% vs. 12.8%, *p* = 0.044). The CHA_2_DS_2_‐VASc score was lower in the <75 years group (4.0 ± 1.4 vs. 5.8 ± 1.3, *p* < 0.001). The other characteristics were comparable between the two groups.

**Table 1:** Baseline characteristics of the study population.

Variables	≥75 years	<75 years	*p* Value
*n*	66	250	‐
Age, years	78.2 ± 4.6	65.4 ± 7.0	<0.001
Female	27 (40.9)	79 (31.6)	0.154
Paroxysmal AF	26 (39.4)	82 (32.8)	0.315
Prior stroke/TIA	42 (63.6)	170 (68.0)	0.502
Prior bleeding	12 (18.2)	53 (21.2)	0.590
Congestive heart failure	15 (22.7)	32 (12.8)	0.044
Hypertension	50 (75.8)	171 (68.4)	0.246
Diabetes mellitus	21 (31.8)	54 (21.6)	0.083
Coronary artery disease	3 (4.5)	13 (5.2)	0.829
CHA_2_DS_2_‐VASc score	5.8 ± 1.3	4.0 ± 1.4	<0.001
HAS‐BLED score	3.1 ± 1.0	2.8 ± 0.9	0.094
LA diameter, mm	42.9 ± 6.0	42.7 ± 6.5	0.769
LVEF, %	61.5 ± 8.0	62.0 ± 7.2	0.662

*Note*: Numbers in the table are presented as mean ± standard deviation or number (percentage).

Abbreviations: AF, atrial fibrillation; LA, left atrium; LVEF, left ventricular ejection fraction; TIA, transient ischemic attack.

### 3.2 Periprocedural data

The periprocedural data are displayed in Table 2. Procedural and fluoroscopy times were comparable between the two groups. Successful PVI and device implantation were achieved in all patients. The Watchman device was the most frequently used, followed by the ACP and LAmbre devices. No patients experienced death or stroke/TIA periprocedurally. Pericardial effusion occurred in 2 (3.0%) and 5 (2.0%) patients aged ≥ 75 years and <75 years, respectively (*p* = 0.639). All patients recovered after pericardiocentesis, and blood transfusion was not needed. The rate of access site complications was similar for patients aged ≥ 75 years and those aged < 75 years (3.0% vs. 4.0%, *p* = 1.000).

**Table 2:** Procedural data.

Variables	≥75 years	<75 years	*p* Value
*n*	66	250	‐
PVI success	66 (100)	250 (100)	1.000
Device implantation success	66 (100)	250 (100)	1.000
Type of device			0.836
Watchman	44 (66.7)	174 (69.6)	
Amplatzer cardiac plug	12 (18.2)	38 (15.2)	
LAmbre	10 (15.2)	38 (15.2)	
Procedural time, min	131.6 ± 22.1	125.4 ± 21.3	0.044
Fluoroscopy time, min	7.5 (6.0, 9.3)	6.5 (5.0, 9.2)	0.019
Echocardiography guidance			0.690
TEE	60 (90.9)	231 (92.4)	
ICE	6 (9.1)	19 (7.6)	
Release attempt, times	1.4 ± 0.6	1.4 ± 0.7	0.508
Adverse events			
Death	0	0	1.000
Stroke/TIA	0	0	1.000
Pericardial effusion	2 (3.0)	5 (2.0)	0.639
Access‐site complications	2 (3.0)	10 (4.0)	1.000

*Note*: Numbers in the table are presented as mean ± standard deviation, median (interquartile range) or number (percentage).

Abbreviations: ICE, intracardiac echocardiography; PVI, pulmonary vein isolation; TEE, transesophageal echocardiography; TIA, transient ischemic attack.

### 3.3 Follow‐up results

**Figure 1:**
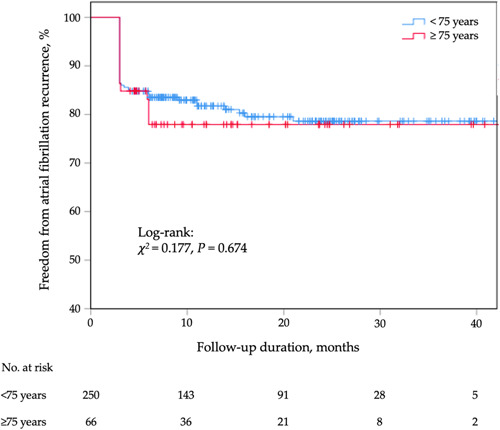
Kaplan–Meier cumulative event‐free curves of atrial fibrillation recurrence.

After a median follow‐up of 12.2 (6.7−24.4) months, 78.8% of the patients aged ≥ 75 years maintained sinus rhythm. In addition, 19.2% of the patients in the <75 years group experienced AF recurrence after 11.9 (5.5−23.6) months of follow‐up (Table 3). Kaplan–Meier curves showed that the rate of AF recurrence was comparable between the two groups (log‐rank test, *χ* ^*2*^ = 0.177, *p* = 0.674; Figure 1).

**Table 3:** Clinical outcomes.

Variables	≥75 years	<75 years	*p* Value
*n*	66	250	*‐*
Follow‐up duration, months			
AF recurrence	12.2 (6.7−24.4)	11.9 (5.5−23.6)	0.365
Clinical outcomes	27.9 (9.3−44.8)	25.2 (10.8−45.7)	0.668
TEE follow‐up			
DRT	1 (1.5)	3 (1.2)	1.000
PDL ≤ 5 mm	9 (13.6)	36 (14.4)	1.000
PDL > 5 mm	0	0	1.000
Death	0	2 (0.8)	1.000
Stroke/TIA	1 (1.5)	4 (1.6)	0.609
Bleeding event	2 (3.0)	13 (5.0)	0.745
Major bleeding	1 (1.5)	8 (3.2)	0.691
Minor bleeding	1 (1.5)	3 (1.2)	1.000
Delayed pericardial effusion	0	6 (2.4)	0.350
AF recurrence	14 (21.2)	48 (19.2)	0.729

*Note*: Numbers in the table are presented as mean ± standard deviation, median (interquartile range) or number (percentage).

Abbreviations: AF, atrial fibrillation; DRT, device‐related thrombus; PDL, peridevice leakage; TIA, transient ischemic attack.

At discharge, 47 patients were taking warfarin, 140 were taking rivaroxaban and 129 were taking dabigatran. DRT was detected in 1 (1.5%) and 3 (1.2) patients among those aged ≥ 75 years and <75 years, respectively. After prolongation of OAC therapy, all DRTs were completely resolved. The percentage of PDL was 13.6% in subjects aged ≥ 75 years, while that in individuals aged < 75 years was 14.4% (*p* = 1.000). No PDL > 5 mm was observed during TEE follow‐up.

**Figure 2:**
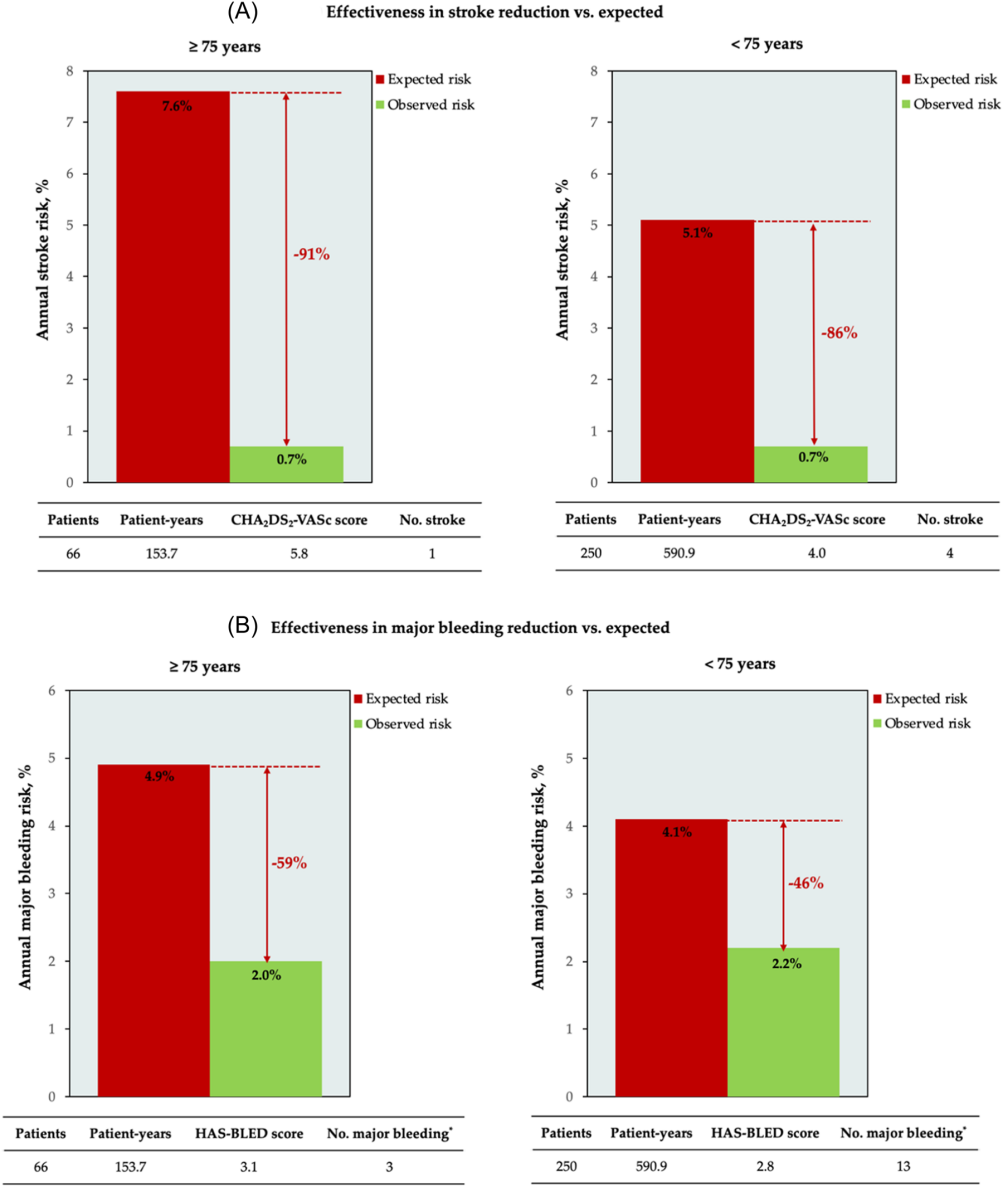
Effectiveness in reducing stroke (A) and major bleeding (B) based on the annual rate predicted by the CHA_2_DS_2_‐VASc score and HAS‐BLED score. Both periprocedural and follow‐up events were included in the analysis.

The median follow‐up periods for clinical outcomes were 27.9 (9.3, 44.8) months in patients aged ≥ 75 years and 25.2 (10.8, 45.7) months in patients aged < 75 years. In patients aged ≥ 75 years, one suffered ischemic stroke (0.7/100 person‐years), and one experienced a major bleeding event (gastrointestinal bleeding). In patients aged < 75 years, four had ischemic stroke (0.7/100 person‐years), and 8 had major bleeding events (six delayed pericardial effusion, 2two gastrointestinal bleeding). No patient aged ≥ 75 years died during the follow‐up period. Two patients aged < 75 years died during follow‐up. One patient died due to infection secondary to atrioesophageal fistula caused by LA ablation 1 month after the procedure. The other patient died due to ischemic stroke 1 month after the procedure. Cerebral CT angiography did not show intracranial artery stenosis. No DRT or significant PDL was detected by TEE. However, patent foramen ovale was found in both preprocedural and repeated TEE examinations, which may be the potential cause of the incident ischemic stroke. DRT or PDL > 5 mm was also not detected in the other four patients experiencing ischemic stroke after the procedure. Two patients finished cerebral CT angiography and severe internal carotid artery stenosis was detected. Including both periprocedural and follow‐up events, the effectiveness of the procedure in annual stroke and major bleeding reduction in patients aged ≥ 75 years were calculated to be 91% and 59%, respectively, while those in subjects aged < 75 years were 86% and 46%, when compared with the expected risk (Figure 2).

## 4 DISCUSSION

### 4.1 Main findings

To the best of our knowledge, the present study was the first to assess the clinical outcomes of combined CA and LAAC in elderly patients with nonvalvular AF. Our investigation proved the safety and efficacy of the combined procedure in elderly individuals.

### 4.2 CA in elderly patients

Recently, the EAST‐AF NET 4 trial demonstrated that AF patients who were randomly assigned to early rhythm control had a lower risk of death from cardiovascular causes, stroke, heart failure hospitalization or acute coronary syndrome versus those assigned to rate control.^11^ Taken to maintain sinus rhythm, AADs can be difficult to manage due to unpredictable metabolism in elderly patients and intolerance of side effects. CA has recently emerged as an important therapeutic alternative to maintain sinus rhythm in patients with AF.^1^ However, the success rate of CA may be affected by a higher degree of atrial myopathy in older individuals.^12^ Randomized trials comparing the efficacy and safety of AF ablation versus medical therapy in elderly patients with AF have not been carried out.

Several nonrandomized investigations regarding CA in elderly patients have been published.^2^, ^3^, ^4^, ^13^, ^14^, ^15^ Willy et al.^2^ evaluated the safety and efficacy of PVI in 146 elderly patients with paroxysmal or persistent AF. Severe periprocedural complications occurred in 3.3% of patients. After a mean follow‐up of 231 ± 399 days, 37.3% experienced recurrence of symptomatic AF. Müller et al.^3^ investigated the efficacy and safety of high‐power short duration ablation in patients over 75 years old (*n* = 120) compared to younger patients (*n* = 420). Overall complication rates were low, and freedom from arrhythmia recurrences was comparable after 12 months (68% vs. 76%, log‐rank *p* = 0.087). Ikenouchi et al.^15^ compared the clinical outcomes of cryoballoon ablation with radiofrequency ablation in elderly AF patients. A total of 198 patients were included and matched by propensity scores. There were no significant differences between the groups in terms of the incidence of complications (cryoballoon 12% vs. radiofrequency 16%; *p* = 0.80) or success rate at 12 months after the procedure (cryoballoon 80.5% vs. radiofrequency 79.4%; *p* = 0.72). Therefore, CA may be safe and effective in elderly patients. However, the inherent limitations of observational retrospective studies do not allow us to draw clear conclusions.

### 4.3 LAAC in elderly patients

Age is a major risk factor for ischemic stroke caused by AF, as highlighted by clinical risk stratification tools designed to predict the risk of stroke in individuals with AF. The CHA_2_DS_2_‐VASc score incorporates age as a major determinant in the prediction of AF‐associated stroke. The most recent ESC guidelines on AF management recommend OACs for stroke risk reduction in individuals with a CHA_2_DS_2_‐VASc score of 2 or greater (Class I recommendation).^1^ Based on this, OACs should be considered in all subjects with AF aged 75 and older, regardless of the presence of other risk factors. However, older age is associated with a greater risk of bleeding complications. A prior study demonstrated that the majority of thrombi originate in the LAA in patients with AF.^16^ Recently, percutaneous LAAC has been proven to be noninferior to warfarin^17^ or direct OACs^18^ for preventing AF‐related stroke. However, there is a lack of data on the safety and efficacy of LAAC in elderly patients.

A single‐center, retrospective observational study included 72 patients undergoing LAAC for stroke prevention, of which 18 subjects were older than 75 years. LAA occluders were successfully implanted in all patients. Periprocedural complications and adverse events during follow‐up were comparable between patients aged < 75 years and ≥75 years.^19^ In a prospective LAAC registry of 638 patients in Germany, 402 (63%) were ≥75 years. The success rate of the procedure was high (97.6%). Periprocedural adverse events occurred in approximately 12%−13% of patients and were not significantly different between age groups (11.9% in <75 years vs. 12.9% in ≥75 years; *p*  =  .80). The 1‐year all‐cause mortality rate was higher in patients aged ≥ 75 (13.0% vs. 7.8%, *p*  =  .04), mainly due to noncardiovascular causes (10.6% vs. 6.0%). No significant differences in major bleeding, stroke, or systemic embolism were observed.^20^ In an LAAO study of 351 patients, no significant differences were found between patients aged < 75 years and those aged ≥ 75 years in total LAAC success rate or procedure‐related major complications within 7 days, all‐cause death, cardiovascular death, stroke/TIA, DRT, or PDL after 2 years.^21^ Therefore, LAAC seems to be feasible and safe in stroke and bleeding risk reduction in elderly patients. However, this needs to be further proven by evidence from randomized controlled trials.

### 4.4 Safety and efficacy of the combined CA and LAAC procedure

Combined CA and LAAC has been practiced for the management of both the symptoms and the high stroke risk of AF.^7^ Combined CA and LAAC procedures were first reported by Swaans et al.^22^ Successful LAAC was achieved in all patients (*n* = 30), with 3 (10%) patients experiencing minor periprocedural complications. At the 12‐month follow‐up, 70% of the patients were free from AF. No thromboembolic events occurred during the 1‐year follow‐up. Calvo et al.^23^ reported a prospective cohort of 35 patients using a mixture of Watchman and Amplatzer Cardiac Plug as a closure device. After a mean follow‐up of 13 months, 78% of patients were free of AF, and the observed ischemic stroke rate was 2.6% per year. Our previous multicenter investigation also proved the safety and efficacy of combined CA and LAAC in the Chinese symptomatic AF population with a high risk for stroke and bleeding.^24^ A total of 122 patients were included. All devices (either Watchman or Amplatzer Cardiac Plug) were successfully implanted, and acute complete LAAC was achieved in 115 (94.3%) patients. The AF‐free success rate without AADs was 76.2% after a mean follow‐up of 11.5 ± 6.8 months. No serious complications were observed during the follow‐up. In the meta‐analysis of Jiang et al.^25^ two randomized controlled trials and 16 observational studies were included. The results showed that combined CA and LAAC in a single procedure has significant efficacy and safety. Taken together, a high procedural success rate with a relatively low complication rate as well as satisfactory follow‐up results were obtained with the combined procedure in the indicated AF patients. Therefore, combined procedure of CA and LAAC could be considered as a potential therapeutic option for symptomatic AF patients with high stroke risk.

However, there were no data regarding combined CA and LAAC in elderly patients. In the current study, we collected and analyzed data from 316 patients who underwent combined CA and LAAC procedures according to age (<75 years or ≥75 years). Periprocedural complications, AF recurrence rate, stroke and major bleeding events were comparable between the two groups, which was consistent with previous investigations. Thus, the results of our research demonstrated the promising safety and efficacy of combined CA and LAAC in elderly patients with AF.

### 4.5 Study limitations

There were several limitations in our study. First, it was a single‐center retrospective investigation. The sample size, especially the number of patients aged ≥ 75 years, was small, which might limit the statistical power to detect differences in some outcomes between the groups. The patients were not randomized, and there could be some confounding factors affecting the outcomes that were not controlled for in the analysis. The follow‐up was done at a single center and might not be representative of the broader population. Second, the data from 24 h Holter monitoring were not available for many patients 1 year after the procedure, leading to a median follow‐up period of 12.2 months for AF recurrence. Therefore, long‐term follow‐up data for sinus rhythm maintenance were lacking in the present study. Third, CA was performed by using radiofrequency energy; thus, the results of our investigation cannot be extended to other types of energy for CA (e.g., cryoablation and pulsed field ablation). Finally, the present study aimed to investigate the clinical outcomes of combined CA and LAAC in the elderly patients and compared with younger patients. Therefore, a control group of elderly patients receiving either CA or LAAC alone was not included, which may limit the ability to compare the effectiveness of the combined procedure with these other therapeutic strategies. Further research is expected to address this point in the near future.

## 5 CONCLUSIONS

Combining CA and LAAC in a single procedure is feasible with acceptable safety and efficacy in elderly patients with AF. Further multicenter, large‐scale, prospective, and randomized controlled trials are warranted to confirm this finding.

## CONFLICT OF INTEREST STATEMENT

The authors declare no conflict of interest.

## Data Availability

The data supporting this study's findings are available from the corresponding author upon reasonable request.
